# Older persons’ experiences of one-to-one in-home support for their digital needs: A qualitative study of a Digital Coach service

**DOI:** 10.1177/20552076251384828

**Published:** 2025-10-03

**Authors:** Elisabeth Berglund Kristiansson, Cecilia Åberg, Anna K. Dahl Aslan, Mia Berglund

**Affiliations:** School of Health Sciences, 7359 University of Skövde, Skövde, Sweden

**Keywords:** Older person, one-to-one support, in-home, digital competence, qualitative study

## Abstract

**Background:**

Older persons (those 65 years of age and older) have the lowest digital competence and are thus at an increased risk of exclusion from a digitalizing society. To bridge this digital divide, several Swedish municipalities have introduced a service called Digital Coach (DC), where older persons can request in-home support for their digital needs. Little is known about older persons’ experiences of this unique service.

**Objective:**

To study older persons’ experiences of one-to-one in-home support, with their digital needs, through the DC service.

**Methods:**

A qualitative study was conducted through interviews, and the data were analysed using qualitative content analysis following the approach of Graneheim and Lundman.

**Results:**

The older persons (*n* = 14) expressed awareness of the importance of digital competence to keep up with the times to avoid exclusion and vulnerability. They requested support to become part of a digital society. The DC service was experienced as providing respectful support at home, which is considered a calm, safe environment. They expressed that the support made them feel valued and increased opportunities for independence and social participation.

**Conclusions:**

Older persons found one-to-one in-home support to be the optimal form of support for their digital needs. The support gave them the best conditions to achieve digital competence, and without the support, would have felt stranded. Efforts to support older persons’ digital competence should be based on the older persons’ own digital needs and be provided in their home.

## Introduction

In today's society, important matters, such as paying bills and booking appointments for health and medical services, are increasingly carried out digitally. In addition, both public and private organizations as well as individuals communicate through digital platforms.^1^ In highly digitalized societies, those without or with low digital competence are at an increased risk of being excluded.^2^ The group with the highest level of digital exclusion is older persons (those 65 years of age and older).^3,4^ However, as new generations enter old age, this is changing. In high-income countries, the majority of older persons have their own digital devices and use the internet daily.^3,5,6^ Still, only about 20–30% of older persons report that they are confident users of digital devices^7^ and never or seldomly need support.^8^ Accordingly, there is need to support older persons in becoming independent in their use of digital devices and internet-based services – a need emphasized by older persons themselves.^9,10^ Interventions and the scientific community have focused mainly on how older persons wish to receive support. Less is known about the experiences of actually getting support, and this is especially about the case with older persons’ experiences of receiving one-to-one support at home.

Digital competence is the knowledge and ability to use the internet and digital technology to support one's work or to fully participate in a modern digital society.^11^ It has been described as a key skill in lifelong learning, providing the ability to participate in networks and to communicate and access information that would otherwise be inaccessible, and thereby increasing the possibility of being independent.^12^ Digital competence includes a combination of knowledge, attitudes, and skills related to digital devices and internet-based services but also the awareness, values, and strategies that make a valuable impact on one's life.^13–15^

Digital competence is lowest among older persons,^5^ although there is great variability between individuals.^10,16,17^ In Europe, about 44% of persons aged 65 years or older are considered to have the competence to manage digital objectives in everyday life.^6^ This also means that more than every second European older person, which corresponds to 50 million individuals, does not have sufficient digital competence. Further, only 20–30% report that they never need help with their digital devices and that they are confident internet users.^7,8^ It is also suggested that digital access and competence in old age are overestimated, as there is a selection bias for healthier and better situated older persons.^18^ The low digital competence among older persons creates a digital divide, which is the gap between those who have access to digital devices and the internet (first level), have the skills to use them (second level), and benefit from this usage (third level), and those who do not. The consequences of the digital divide make social inequalities.^19^

There is a broad range of reasons for why older persons have lower digital competence and experience the digital divide, but physical and cognitive factors are identified as major barriers.^20,21^ For some older persons, the cost of digital devices and a lack of devices limit possibilities to practice and achieve digital competence.^22^ In addition, many older persons worry about making errors^10^ or describe a lack of motivation to learn.^20,21^ A common barrier is not having anyone to ask for guidance and support.^10,21^

To a large extent, older persons have to rely on informal support (e.g. family and friends),^10,23^ but there is a growing body of formal initiatives to support older persons’ achievement of digital competence (e.g. classes and coaches).^9,10^ The majority of initiatives to support older persons’ digital competence have so far been group-based education.^9^ The advantages of group-based education include that it is efficient economically and time-wise. At the individual level, group-based education might be beneficial in a familiar atmosphere, so that participants are able to ask questions, learn from each other, and practice together.^24^ Social support is reported to be important for achieving digital competence, however, a disadvantage of group-based education is heterogeneity among group members with regards to needs and previous knowledge. This creates difficulties both for the group members and for the instructor.^9^ Shame or a lack of confidence in not knowing or understanding enough about digital matters also affects one's ability to learn in a group.^9,25^

Another form of support to increase digital competence is one-to-one support. This form is often suggested when older persons are interviewed about how they want to achieve digital competence.^17,25^ One-to-one support is thought to make it possible to better address questions and problems, which are henceforth referred to as digital needs. In this support, the pace can be adjusted.^25^ Furthermore, one-to-one support should include the possibility to practice,^17,25^ as older persons want to learn how to solve problems by themselves and acquire so-called know-how knowledge.^23^ One-to-one support is also thought to increase motivation both to start and to continue using digital devices but also to overcome barriers such as fear or shame over not having sufficient digital competence.^9,25,26^ Despite studies largely concluding that older persons want personalized support, most interventions are group-based and performed in public spaces.^9^

To the best of our knowledge, most previous studies of one-to-one support lack elements that the current study consists of, that the support is based on older person's own digital needs provided at home. However, there are some previous studies of one-to-one support, albeit short term. An example is a pilot study by Arthanat et al., in which older persons were given access to a tablet (which they previously did not have) and in-home support by a graduate occupational therapy student with time allocated for teaching and asking questions about the tablet.^27^ The study's findings suggest that the older persons gained a more positive attitude towards digital technology and increased independence in the use of digital devices. A follow-up study showed that the participants experienced benefits from their digital training, such as the ability to use online healthcare resources or read newspapers digitally.^28^ However, the study also reported that the older persons found it challenging to maintain the knowledge they had gained from the digital training because there was no possibility of getting ongoing support.

In Sweden, an increasing number of municipalities are offering support to diminish the digital divide among older persons. In some, this support is provided one-to-one in the older person's own home by a municipality-employed civil servant. The service is called Digital Coach (DC; in Swedish, *Digital fixare*) and is intended to increase older persons’ digital competence by addressing their digital needs. The support can include information and advice about using digital tools and common digital services, installing applications, and creating accounts. To the best of our knowledge, this is a novel form of support provided at home that has not yet been studied. Therefore, the research aim was to study older persons’ experiences of receiving one-to-one in-home support (i.e. DC) for their own digital needs.

## Methods

### Study design

An inductive qualitative approach was applied, based on qualitative interviews with older persons to gain information about their experiences of individual in-home support with their own digital needs via the DC service. The interview data were analysed using qualitative content analysis method according to Graneheim et al..^29^ The study was guided by the consolidated criteria for reporting qualitative research checklist.^30^

### Setting

The study was conducted in a medium-sized Swedish municipality between June and December 2021. In December 2020, the municipality was one of the first to offer the DC service. It was initiated during the COVID-19 pandemic to bridge the digital divide and combat involuntary loneliness. The service was also something that seniors’ organizations had requested from the municipality. The service is free of charge, has no restrictions on the number of visits, and is available to all citizens aged 65 years or older in the municipality. The DC service is entirely run by the municipality and not one provided by the researchers. At the time of the study, there was one DC in the municipality. Currently, there are two full-time DCs, both of whom are nursing assistants with training in welfare technology. Recent information from the national network of DCs shows that around 33 of Sweden's 290 municipalities offer some kind of DC service, and the number of employees amount to approximately 45 persons with varying backgrounds.

An older person contacts the municipality and asks for an in-home visit by a DC. After that, the DC contacts them to jointly decide on a time. Two hours are commonly scheduled, and the support is designed to be based on the older person's individual digital needs. During a typical visit, the parties introduce themselves before they enter a room where the older person has prepared a list of digital problems they face and laid out any digital devices they own. The conversation is usually initiated by the older person, who talks about their digital needs. Examples of what the DC service offers are in the use of mobile phones, computers, televisions and their functions, installing and getting started with applications, and advice about the internet and digital services. During the visit, the DC focuses on addressing the older person's specific digital needs. At times, the DC also gives examples of digital devices that may be useful for the older person, such as those offering larger text for increased readability.

### Data collection, participants, and procedure

Before participants were recruited for the study, contact was established with the municipality's public health department, which coordinated dialogue with the DC, the municipality's Information Technology department, and the researchers. None of the researchers knew either the DC or the participants before the start of the study. The DC was informed both orally and in writing about the study and that participation was voluntary. Inclusion criteria for participating were persons aged 65 years or older who had contacted the municipality to receive support from the DC. The DC asked older persons if they were willing to participate in the study when the DC and the older person decided on a time for the in-home visit. The selection of participants was based on convenience sampling.^31^ All older persons who contacted the DC spoke and understood Swedish, as the DC service is provided in Swedish. In the recruitment process, a balanced representation of persons in-town and in the suburbs was pursued. No other selection or exclusion criteria were used.

Fourteen persons were invited to participate in the study, and all agreed to participate. The number of participants was decided before the interviews were conducted, as it was considered sufficient to meet the aim. The amount of data necessary to answer a research question in a credible way varies depending on the complexity of the phenomena under study and the quality of the data.^32^ Saturation was assessed using the concept of information power, which considers factors such as study aim, sample specificity, dialogue quality, and analysis strategy. It focuses on the information richness of the interviews rather than saturation based on the number of interviews.^33^

After the initial invitation from the DC to participate in the study and an affirmative answer from the respective participants, the researcher contacted the older persons and informed them about the study. The researcher's first visit to the older person's home was during the DC's in-home visit. The visit times occur when both the DC and the researcher were available to visit the older persons’ home, with the researcher participating only as an observer during this visit. Data collection for this study, however, was carried out later by the researcher without the DC present. The researcher carried out non-participant observations; data from these are not included in the present analysis and will be presented in a separate publication. The interview was conducted in the older person's home 2 to 3 weeks after the DC had provided support to the older person. The DC was not present during the interviews. The procedure, to first visit together with the DC and then return to do the interview, facilitated the researcher's understanding of the support meeting and meant that the older person recognized the researcher.

The interview guide was constructed by the authors, of whom the third and last authors are senior researchers with extensive experience in research methods, while the first author is a PhD student with a background as a registered nurse and a teacher in public health at university, and has experience using interviews to collect data. The questions in the interview guide were developed by these authors following a framework for qualitative semi-structured interview guides,^34^ based on the research aim, a literature review, and discussion.

The interviews, which were collected by the first author, were based on the interview guide, which included open questions related to the aim. The researcher started the interview with the following: ‘How was it to receive support from the DC at your home?’ Follow-up questions were asked, such as: ‘What and why was support needed?’ For further details, see Supplemental 1. This approach gave the participants the opportunity to develop their answers from their own perspectives.^35^ The interviews were audio-recorded and typically concluded when the researcher felt that the topic had been thoroughly addressed, with responses supported by the questions in the interview guide.

### Data analysis

The interviews were transcribed verbatim and analysed using inductive content analysis according to Graneheim and Lundman.^29,32^ The first author read the transcribed text several times to get a sense of the whole, and meaning units relevant to the study's objective were identified. These units were then condensed, and each was assigned a code as shown in Table 1. The codes were compared and grouped for similarities and differences and sorted into subcategories, which in turn were abstracted into categories. Within the categories and subcategories, the level of abstraction varied; at the most concrete level are quotations, while the underlying meaning of the text was interpreted and emerged in the theme, which was at the latent level. Every step in the process was discussed several times by all of the co-authors, and the original text as well as the text of the various analysis steps were read individually and together to reach a common understanding of interpretations. The analysis was an iterative, back-and-forth process with constant interaction between the data and their interpretation.^36^

**Table 1.** Example of the qualitative content analysis process.

Meaning unit	Condensed meaning unit	Code
… you’re a little unsure when you think everything will disappear [from the computer] … But it didn’t, [DC] explained. … It's relieving in some way …	are unsure … think everything disappears from the computer … DC explains and calms you down	Understanding reduces uncertainty

DC: Digital Coach.

### Ethical considerations

The present study was conducted in accordance with the ethical criteria of the World Medical Association Declaration of Helsinki: Ethical Principles for Medical Research Involving Human Subjects^37^ and approved by the Swedish Ethical Review Authority (DNR 2021-03524). Each participant was informed before the process, both in writing and orally, about the study and how it would be conducted. They were also informed that participation was voluntary and that they could withdraw at any time without explanation or consequences. Before the DC support began, verbal and written informed consent was obtained from the participants to be interviewed and audio-recorded. To protect the privacy of the participants, names have been coded.

## Results

### Description of the participants

The sample comprised of 14 participants between the ages of 68 and 92 years. Six were female and eight males. Their place of residence was either in town or just a few kilometres outside it. Ten participants lived alone and four with their partner (Table 2). The transcribed material was based on the interviews, which lasted between 40 and 70 min.

**Table 2.** Demographic data of the participants.

	Female	Male
Gender	6	8
Lived alone	6	4
Lived with a partner	-	4
Lived in town	4	4
Lived in a suburb	2	4

At the initial stage, there were more than 100 codes, which were reduced and formed 16 subcategories, which finally resulted in six subcategories, three categories, and one theme. The theme, ‘To be a valued person in the digital society with a need to keep up with the times’, captured underlying meanings, assumptions, and ideas that were not explicitly stated but were interpreted from the data. The three categories are descriptive groupings of content that shares a common theme and reflects the manifest meaning of the text. The six subcategories break the categories down into more detailed and specific aspects, helping to clarify nuance or variation within a broader category. The categories are close to the data in that they include examples and citations. The theme, categories, and subcategories are presented in Table 3, and described in detail below.

**Table 3.** Overview of theme, categories, and subcategories.

Theme	To be a valued person in the digital society with a need to keep up with the times		
Category	To manage everyday life in the digital society	Receiving respectful individual support	Digital competence empowers autonomy and well-being
Subcategory	Willingness to manage digital needs despite challenges (a) To not be vulnerable and excluded (b)	Support based on personal needs (c) Home offers a calm and safe environment (d)	Increased independence and satisfaction (e) Enables social participation (f)

### Theme

Older persons’ experiences of receiving individual in-home support for their digital needs through the DC service appear in the theme ‘To be a valued person in the digital society with a need to keep up with the times’. Being a valued person means having one's own needs met and being respected for who one is, by receiving respectful one-to-one support. The ability to handle digital devices and services independently after the support brought joy, satisfaction, and greater opportunities for social participation and independence, along with the feeling of keeping up with the times, which is considered a necessity.

### Categories and subcategories

#### To manage everyday life in the digital society

The older persons express that it is a necessity to have digital competence to manage everyday life in today's society. This involves understanding and being able to manage digital devices, applications, and internet-based services. All expressed a willingness to achieve more knowledge, despite this being more or less challenging. To facilitate achievement of digital competence and avoid feelings of not being competent and the associated shame, the older persons emphasized the necessity of one-to-one support. This category encloses subcategories a and b (Table 3).

##### Willingness to manage digital needs despite challenges

A willingness to manage digital needs became apparent, as the older persons expressed that their primary reason for contacting the DC was to independently handle their digital devices and internet-based services. Specifically, they express that they asked for help, as they wanted to manage a television, computer, printer, tablet, or mobile phone without help. They also expressed that they needed help with applications, like BankID, a Swedish digital identification tool broadly used to identify oneself, and with internet-based services. The associated language was described as difficult to understand, which was another reason the older persons needed support. For example, they expressed that they do not have enough pre-knowledge to understand written instructions. Several expressed that the complexity was a challenge, that one needed to know and be able to perform many things before one is able to use the digital device, for example when a new smartphone is bought.

… had … bought this new iPhone, and then there were various things that you had to put in there, that Apple ID and stuff … And passwords and one thing and another one came up … also wanted BankID, and … Swish, you know … (Participant 9).

Some described the lack of pre-knowledge as overwhelming and how they sometimes wanted to give up. One older person expressed this as a feeling of resignation and a loss of faith in their own abilities. Still, the willingness to continuously manage digital needs was described as something important to maintain.

… I hear some say, ‘I don’t care about my invoices. My son does that.’ That is to declare yourself incompetent … (Participant 3).

##### To not be vulnerable and excluded

The experience of having insufficient knowledge and understanding of digital devices and internet-based services was described as contributing to feelings of being vulnerable and excluded from the digital society. It also created uncertainty and worries, for example of losing everything on the computer (such as documents and pictures). The older persons described the support from the DC as making them feel less vulnerable, for example by helping them make backups or confirm that everything was in order.

… you’re a little unsure when you think everything will disappear … [from the computer] … But it didn’t. [The DC] explained. … it's relieving in a way … (Participant 2).

They also described feelings of shame and fear regarding their inability to handle digital matters themselves. They faced a dilemma: on the one hand, they were reluctant to ask for support because they felt it would be overwhelming and humiliating; on the other hand, they were afraid to try things on their own due to the possibility of failure. Some older persons describe that the consequences sometimes prevented them from doing things they enjoyed, such as watching a desired movie.

They expressed that the DC gave them confidence by explaining and supporting them in addressing their digital needs. Examples included fear about doing something wrong when installing a new computer or when advertisements come up on their phones.

… when I called, I wanted to find out how to clean my computer … I received advertisements on my phone then on Facebook … so I thought that there must be something wrong … I thought then [the DC] could, if they would remove [them] … And I got an answer to that (Participant 5).

Receiving an answer or an explanation or getting a problem solved was experienced as reducing the feeling of being vulnerable and excluded.

#### Receiving respectful individual support

Receiving respectful individual support meant that the support was based on one's own digital needs and being respected for who one is and the situation one finds oneself in. The older person's own home was experienced as a calm and safe environment to expose needs and knowledge gaps related to their digital devices and internet-based services. Compared to other forms of support that they had encountered, the experience of one-to-one support in their own home was referred to as the optimal form of support. This category encloses subcategories c and d (Table 3).

##### Support based on personal needs

The older persons describe that when they found themselves being listened to and considered in their current situation, this was experienced as receiving support based on personal needs. They shared examples of personal needs related to health changes, such as impaired hearing and eyesight, as well as weakened memory and learning ability. The DC was described as taking their physical and psychological limitations into consideration. The DC was also said to adjust support to their previous digital experiences and to the digital devices they possessed. They found themselves being listened to and felt that the DC started from their needs and taught them step by step.

Additionally, the DC not only addressed the problems that they explicitly noted but also paid attention to what was discussed and described during the ongoing support. The DC thereby addressed problems that were not explicitly stated.

The older persons generally prepared their questions before the visit. One described that the best support for learning to handle digital devices was when the DC first gives instructions, then the older person did exercises with the DC and, finally, practiced by themselves in peace and quiet. Others said that it was easier to remember something if they practiced immediately after the DC left. Examples were given where the DC assigned them tasks or challenges, such as to take a photo and send it to someone. The older persons express a wish to be supported on a recurring basis, as they thought that would support their digital competence.

… you’re toying with the idea that … [the DC] will now come back several times then I’ll get to work on it more … (Participant 7).

It was important to the older persons that the DC did not provide support that was too extensive or given too quickly. Additionally, they noted their own responsibility to not bring up all their digital needs at once, as some experienced it becoming overwhelming, which made it difficult to absorb the information.

… I would just take these two problems, the one with the TV and then the computer … I wouldn’t have taken anything else. I had a long list I’d written; there was so much to go through, and like, we got tired, and I forgot anyway …  (Participant 1).

Sometimes, it was described that the DC solved a practical problem, and the older person did not learn or did not want learn how. This was described as a one-off solution, for example, setting a larger font size on a mobile phone or setting up antivirus software, and a necessity to being able to use their digital devices and benefit from the internet.

The older persons often compared the DC support to other forms of support they had previously sought, for example from computer companies or relatives. They expressed that staff at computer companies did not provide age-adjusted support. For example, they went too fast, which made the older persons feel patronized. Support from children and grandchildren was described as helpful, but they might not have enough time to support their older relative or live far away. Support from the DC was considered optimal.

##### Home offers a calm and safe environment

In-home support was experienced as safe and the best environment to get support. When support is provided in a calm environment and by a calm person, the older persons explained that they felt relaxed and comfortable asking questions. Being in their own home enabled them to focus on their own digital needs without interference from others. This was emphasized as being especially important in old age.

I just can’t do that much … because I have … [a lung disease] … if I overexert myself, I get dizzy and just want to throw up. So, I’m actually mostly at home, and then it's even more important that you have … this … support … (Participant 11).

They also compared in-home, one-to-one support with group-based support in a public place, where the latter was described as messy and it felt impossible to learn and remember anything – as described by one older person who had previously taken part in group-based support:

The security token [in Swedish, *bankdosa*] was new, that was enough. I didn’t understand anything. Then we were a group of over 15 people I think, and everyone was talking over each other. It was mixed up (Participant 2).

Some said that some issues are sensitive and better handled at home, otherwise it felt insecure. Therefore, in-home support was preferred.

#### Digital competence empowers autonomy and well-being

Digital competence empowers autonomy and well-being, which implies freedom for older persons to continue to have control over their own lives. The older persons expressed satisfaction achieving increased digital competence, as it provided the opportunity to independently use their own digital devices and share experiences with others. This contributes to fostered feelings of joy and happiness. This category encloses subcategories e and f (Table 3).

##### Increased independence and satisfaction

When the older persons developed their digital competence, with the support of the DC, their feelings of independence and satisfaction increased. For example, managing digital personal identification independently was described with pride.

When I spoke to [the insurance company] the other day, [they asked] ‘Now I need to know that it is you who is calling. Do you have a BankID?’ Yes! I did! (Participant 3).

Regardless of whether it involved important digital matters, such as banking, or everyday tasks such as buying clothes or food online, it was satisfying to do so without asking anyone for support. The DC support was expressed as enabling feelings of joy and freedom.

… deep gratitude that the DC did it. But now that all the knots are untied and all the problems are out of the way and stuff, this is when I think my life begins …  (Participant 11).

The older persons expressed that support for their digital needs was crucial for their independence, as other types of assistance provided by society supported older persons remaining in their own homes.

… I have to say that I’m so terribly satisfied and grateful for this support that you can get because this is what we older persons need most. I mean, we can get help with cleaning and things like that, we do that easily, but it's not that easy to get good support with IT issues (Participant 8).

##### Enables social participation

It was expressed that the support enhanced social participation. This encompasses the ability to maintain communication with close relations via digital devices but also receiving information about digital aids. Email was considered an important resource to have access to. Furthermore, knowledge about how to deal with digital photography was considered important for participation, as it was a way of sharing experiences and feeling happiness with others. For some who received support using social media, it was described as a long-awaited event to be able to follow the grandchildren via free applications.

It goes under Messenger … Yes, great, great fun. The little one … had been given a trampoline … ‘Old grandma, look now Old grandma, now I’m jumping’ … I think I sat for 20 minutes hee hee … (Participant 2).

Learning to communicate digitally was described as allowing possibilities for new ways of contacting others and reducing the risk of being isolated.

I mean, if I hadn’t gotten this support, I wouldn’t have been able to, I probably would have become quite isolated … (Participant 11).

It was especially meaningful and valuable to get support from the DC to be able to keep in contact with children, grandchildren, and other relatives.

Social participation was further enabled when the DC suggested using headphones to hear better. The older persons said they no longer hesitated to answer the phone or call someone, which had been a problem for those with hearing problems.

## Discussion

The current study was focused on older persons’ experiences of DC, a novel, in-home support service where their digital needs (questions and problems) in relation to their own digital devices and internet-based services were at the core of the support. The older persons who had requested and received the support, valued the opportunity to receive individual, respectful, in-home support. There was an awareness among the participants that they had to keep up with the times, which meant that they had to achieve or extend their digital competence despite this sometimes being experienced as challenging. To move forward and overcome barriers, the DC support was referred to as a necessity. Achieving digital competence and moving past barriers contributed to their independence and enabled their social participation. In-home, one-to-one support was described by the participants as an optimal form of support with their individual needs in old age.

The digital gap of digital competence, mentioned as digital divide, is described in three levels: first level – those who have access to digital devices and the internet, second level – have the skills to use them, third level – and benefit from this usage, and those who do not.^19^ The current study confirms that there is a group of older persons that understands they need, and have to take an active role to achieve digital competence to manage everyday life in a digital society, which refer to third level of digital divide. However, they are often hindered by not having enough skills or confidence in their own ability (second level of digital divide), as others have also shown.^5,21^ Without having someone to ask for support, either in a one-off situation or in achieving the knowledge and understanding to perform independently, these older persons feel that they cannot take an active part in society. In other words, they understood the benefits of internet use and being digital, but they are unable get through the second level of the digital divide by themselves.

A core feature of this in-home one-to-one support is that plenty of time is set aside, 2 h, so there is no need to rush, either for the older person or for the DC. Sufficient time, so that the DC could listen and adapt the support to their individual digital needs and situation, was appreciated by the older person. Adapted support has been reported to strengthen older persons’ learning.^38^ It can only be speculated, but it is likely that the DCs background as an assistant nurse contributed to an understanding of how to interact with older persons and thereby provide support adapted to their physical and psychological challenges.

The older persons described different approaches to achieving digital competence that they had found useful or would find useful in the future, and these ranged from getting things explained and watching to practice both individually and with support, or afterwards. Strategies like writing down questions beforehand and taking notes during the coaching were also described. Another observation was that it might be more successful to not aim to address all the questions in a single session, as it becomes overwhelming. The heterogenous learning preferences observed could in future interventions be addressed by asking the older persons about their learning preferences.

The home environment was expressed to be the best place to receive support, because many digital matters are sensitive, for example the creation of passwords, and at home the risk of someone overhearing the conversation is absent. The security aspect is a strong argument for the need for one-to-one support in a secure location. Further, as the attention span tends to decline in older age,^39^ a calm environment increases the chance that an older persons is able to focus, which is especially important when something is perceived complex and challenging, such as achieving digital competence. This might be one reason why the older persons often compared the DC service to group-based interventions they had participated in and found challenging. Another benefit of receiving support at home is that the older person can ‘save their energy’ instead of spending it on travel to get support.

One common motivation to ask for support was the desire to maintain social relationships, especially with children and grandchildren, and this could be facilitated by creating a social media account, learning how to send pictures, or using headphones, which made it possible to hear better. As motivation is a valuable driving force, the ability to maintain social relationships through increased digital competence is a strong argument that needs to be used when supporting digital needs. This particularly important with those who are not convinced they need digital competence, as others also have suggested^38^ – not least since close relationships with family members are seen as the most important aspect of well-being in later life.^40^ It can be hypothesized that strengthened digital competence can be one method to counteract loneliness in old age, but this remains to be explored.

Further, the aspect that many older persons expressed pride and joy in being able to manage digital devices, applications, and internet-based tasks themselves should be emphasized, as overcoming digital barriers can be understood to contribute to their well-being. As mentioned in the Results, one even expressed that it was now that his life began.

Hence, stakeholders, such as municipalities, civic organizations, practitioners, and researchers, need to design and to develop interventions that address this new generation of older persons’ digital needs. Specifically, there is a need for more in-home, one-to-one support, as it is considered the optimal form of support. This confirms results from previous studies where older persons were asked *how* they wanted to receive support with their digital needs.^17,25^

A strength of this study is the rich number of experiences collected through the interviews. This was especially valuable, as both interventions and studies about one-to-one, in-home digital support are limited. Furthermore, the researcher followed up on what was said to enable a deeper understanding of what was requested. Another unique aspect is that the interviewer was present and observed when the DC support was provided. On the other hand, this might have affected what emerged in the interview and the researcher's pre-understanding. The researchers were aware of this risk; therefore, the older persons were encouraged to talk about their experience with the support. Additionally, throughout the analysis, the question ‘Did this emerge in the interviews’ was present, which forced a process of reflection and returning to the text.

Another part of trustworthiness is transferability, which in this study, participants were convenience sampled and included those who had made initial contact with the DC themselves. We do not claim that the findings are representative of all older persons, but they are likely transferable to older persons who want support to extend their digital competence. Another concern is that the older persons all received support from the same DC, a DC known to be appreciated by them. This may have affected the results and indicate that this particular DC acted in a certain way and possessed certain knowledge, which could have steered the outcomes of the interviews in a certain direction. On the other hand, the study included 14 older persons who did not know each other and who had different digital needs and, therefore, contributed to several novel findings that emerged in the results.

Finally, an important and fragile part of a study is the analysis. Therefore, in this study to ensure trustworthiness, specifically dependability, the phases of decontextualizing and recontextualizing in the qualitative content analysis needed to be strengthened. This means that each step in the analysis was carefully handled and frequently discussed by the co-authors, and as these steps were followed in collaboration, the dependability of the analysis is expected to be strengthened.

## Conclusions

There is a need for support to increase the digital competence of older persons, and in this study, they were aware of this and expressed a willingness to gain access to it. They felt that the optimal form of support was one-to-one in their own home, because the support focuses on their digital needs, as home is experienced as safe and calm, without distractions from noise and other people. Large benefits of using digital devices and important reasons for requesting in-home support were the older persons’ desire to keep in contact with family and friends and to be independent. Becoming more able in these domains seemed to contribute to their well-being. Therefore, a larger number of interventions to increase older persons’ digital competence should be personalized and performed in the home. Future studies should explore whether increased digital competence is a means to counteracting loneliness. Moreover, as the study highlights the importance of one-to-one in-home digital support to help older persons develop digital competence, further studies are recommended to investigate how similar interventions are implemented in other countries around the world. It would also be interesting for the future studies to investigate whether there are alternative models to one-to-one support that can be equally effective in supporting older persons digital competence in the digitization process.

## Supplemental Material

sj-docx-1-dhj-10.1177_20552076251384828 - Supplemental material for Older persons’ experiences of one-to-one in-home support for their digital needs: A qualitative study of a Digital Coach service Supplemental material, sj-docx-1-dhj-10.1177_20552076251384828 for Older persons’ experiences of one-to-one in-home support for their digital needs: A qualitative study of a Digital Coach service by Elisabeth Berglund Kristiansson, Cecilia Åberg, Anna K. Dahl Aslan and Mia Berglund in DIGITAL HEALTH

sj-pdf-2-dhj-10.1177_20552076251384828 - Supplemental material for Older persons’ experiences of one-to-one in-home support for their digital needs: A qualitative study of a Digital Coach service Supplemental material, sj-pdf-2-dhj-10.1177_20552076251384828 for Older persons’ experiences of one-to-one in-home support for their digital needs: A qualitative study of a Digital Coach service by Elisabeth Berglund Kristiansson, Cecilia Åberg, Anna K. Dahl Aslan and Mia Berglund in DIGITAL HEALTH
