# Supplementary material for: Older persons’ experiences of one-to-one in-home support for their digital needs: A qualitative study of a Digital Coach service
Source: Digit Health. 2025 Oct 3;11:20552076251384828. doi: 10.1177/20552076251384828 (PMC12495190; doi:10.1177/20552076251384828)
Supplement: sj-docx-1-dhj-10.1177_20552076251384828 - Supplemental material for Older persons’ experiences of one-to-one in-home support for their digital needs: A qualitative study of a Digital Coach service [file sj-docx-1-dhj-10.1177_20552076251384828.docx]

### Interview guide – “Older persons’ experiences of one-to-one in-home support with individual digital needs: A qualitative study of the

### Digital Coach service”

Start question: How was it to receive support from the DC at your home?

1. What did you need support with? Why was support needed with it?
2. Describe what it was like to get support from the digital coach in your own home?
   1. How did you experience the support? (Describe as concretely as possible)
   2. What did you think and feel in the situation?
      - Ex Was there anything that hindered the support?
      - Ex Was there anything that made it easier?
3. Did you learn anything? If so, what? When? (When did you understand?)
   1. What made you learn (for example, how to email?)
   2. What was difficult?
4. How have you used your new knowledge?
5. What significance has the support of Digital Coach had for you? (for example, more participation, more social contact)
6. Have you previously received support with digital tools? How did it go? How did you experience it?

Follow-up questions: what happened, how did you think, how did you feel, why, describe a little more, when.
